# Kaempferol Protects Cell Damage in In Vitro Ischemia Reperfusion Model in Rat Neuronal PC12 Cells

**DOI:** 10.1155/2020/2461079

**Published:** 2020-04-23

**Authors:** Ya-ping Zhou, Guo-chun Li

**Affiliations:** School of Medicine & Holistic Integrative Medicine, Nanjing University of Chinese Medicine, Nanjing, Jiangsu 210023, China

## Abstract

Ischemic cerebral stroke is a severe neurodegenerative disease with high mortality. Ischemia and reperfusion injury plays a fundamental role in ischemic cerebral stroke. To date, the strategy for ischemic cerebral stroke treatment is limited. In the present study, we aimed to investigate the effect of kaempferol (KFL), a natural flavonol, on cell injury induced by oxygen and glucose deprivation (OGD) and reoxygenation (OGD-reoxygenation) in PC12 cells. We found that KFL inhibited OGD-induced decrease of cell viability and the increase of lactate dehydrogenase (LDH) release. OGD-induced activation of mitochondrial dysfunction, mitochondrial apoptotic pathway, and apoptosis was inhibited by KFL. KFL also reduced OGD-induced oxidative stress in PC12 cells. P66shc expression and acetylation were increased by OGD and KFL inhibited these changes. Upregulation of P66shc suppressed KFL-induced decrease of apoptosis, the decrease of LDH release, and the increase of cell viability. Furthermore, KFL inhibited OGD-induced decrease of sirtuin 1 (SIRT1) expression and downregulation of SIRT1 blocked KFL-induced decrease of apoptosis, the decrease of LDH release, and the increase of cell viability. In summary, we identified that KFL exhibited a beneficial effect against OGD-induced cytotoxicity in an ischemia/reperfusion injury cell model. The findings suggest that KFL may be a promising choice for the intervention of ischemic stroke and highlighted the SIRT1/P66shc signaling.

## 1. Introduction

Ischemic cerebral stroke is a severe neurodegenerative disease that accounts for approximately 70–80% of all cerebrovascular patients with a high risk of disability and mortality [1]. There are approximately 15 million ischemic stroke patients each year which leads to 5 million deaths annually [2]. Ischemic stroke is characterized by the sudden neuronal death in the brain due to ischemia and reperfusion injury. Various pathological processes are involved in ischemic cerebral stroke, including oxidative stress, apoptosis, inflammation, neuronal excitotoxicity, and disequilibrium of energy metabolism [3]. These conditions interact and overlap, forming a vicious cycle and resulting in irreversible and persistent dysfunction neurology.

A primary concern for reducing ischemic stroke injury is to recover cerebral blood flow and oxygen supply. However, the resultant cerebral ischemia and reperfusion injury may further aggravate pathological damage in the ischemic district which is potentially rendered irreversible [4]. Tissue plasminogen activator (tPA) is the only approved drug by the US Food and Drug Administration for the treatment of stroke [5]. tPA mainly functions to dissolve the clots of blood and restore the blood flow to the brain. This treatment may be limited by the aftermath of following reperfusion injury [1]. Moreover, tPA treatment is only suitable for less than 10% stroke patients (<10%) [6]. However, there are no other novel strategies that have proven to be efficacious and safe for the clinical intervention of ischemic [7].

Kaempferol (3,4 0,5,7-tetrahydroxyflavone, KFL), a flavonol, is a natural product that could be extracted from plenty of natural sources, including strawberries, Ginkgo biloba leaves, Pu-erh tea, paprika, and butterbur (Petasites japonicus) [8, 9]. It has been reported that KFL possesses a variety of biological activities, including antitumor, antidiabetic, anti-inflammatory, and antioxidant effects [10, 11]. In particular, KFL has been shown to exhibit neuroprotective activities. For instance, Kim et al. have shown that KFL and its derivatives could enhance cognitive activities [12]. Yu et al. showed the neuroprotective effect of KFL glycosides against brain injury and neuroinflammation in transient focal stroke [13]. López-Sánchez et al. found that blood micromolar concentrations of KFL could afford protection against ischemia/reperfusion-induced damage in rat brain [14]. However, the mechanism of potential protective effects of KFL against ischemic cerebral stroke is not clear. In the current study, we designed experiments to investigate the mechanism of KFL-induced effect on ischemia and reperfusion injury in PC12 cells.

## 2. Materials and Methods

### 2.1. Reagents and Chemicals

KFL was obtained from Sigma-Aldrich (Shanghai, China). Antibodies against Sirtuin 1 (SIRT1), P66shc, Ac-lysine, Cyclin-dependent kinase 4 (CDK4), CDK6, cleaved caspase 3, caspase 9, caspase 7, PGC-1*α*, NRF-1, COX-IV, and *β*-actin were purchased from cell signaling technology (Danvers, MA, USA).

### 2.2. Cell Culture and Differentiation

PC12 cells were purchased from American Type Culture Collection (ATCC, Manassas, VA, USA). PC12 cells were cultured in Dulbecco's Modified Eagle Medium (DMEM) (Life Technologies, Carlsbad, CA, USA) supplemented with 10% horse serum (HS, Life Technologies, Carlsbad, CA, USA), 5% fetal bovine serum (FBS, Life Technologies, Carlsbad, CA, USA), and 1% Penicillin/Streptomycin (P/S, Life Technologies, Carlsbad, CA, USA) at 37°C with 5% CO_2_ in an incubator.

For the induction of differentiation, PC12 cells were seeded onto poly-l-lysine-coated petri-dishes. 24 h after the plating of cells, the medium was replaced with serum-free differentiating medium (DMEM, supplemented with 1% penicillin-streptomycin, 100 ng/ml Nerve growth factor (NGF)). The differentiating medium was replaced every two days for a period of 7 days. At last, the cells completely transformed into differentiated neuronal cells.

### 2.3. OGD Treatment

The treatment of oxygen and glucose deprivation (OGD) and reoxygenation (OGD-reoxygenation) was established as previously reported [15]. The culture medium was removed and cells were cultured in glucose-free Earl's balanced salt solution (EBSS) and in an environment with 95% N_2_ and 5% CO_2_ at 37°C. After 4 h glucose and oxygen deprivation, cell culture medium was replaced with normal medium and cells were transferred to the normal incubator for 24 h. The indicated concentrations of KFL were added to the culture medium during the entire period of OGD-reoxygenation. For lentivirus-mediated infection, cells were transfected with LV-P66, LV-shSIRT1 or their negative vectors. After purification with puromycin, stably transfected cells were exposed to OGD-reoxygenation in the presence or absence of KFL.

### 2.4. Cell Viability

In brief, 6 × 10^3^ cells per well were seeded in 96-well plates. After the treatment of OGD-reoxygenation and KFL, the medium was replaced with fresh serum-free DMEM medium. 10 *μ*l WST-8 solution (Beyotime Biotechnology, Shanghai, China) was added into a well of 96-well plates. Then, the plate was incubated 37°C for 1-2 h. After that, the absorbance was measured by spectrophotometry at 460 nm using an ELISA reader. The results of cell viability were expressed as the fold-change of control.

### 2.5. Lactate Dehydrogenase (LDH) Release

After the experiment, the culture medium was collected and LDH content in the medium was assayed using LDH assay kit (Bio Vision Inc., Milpitas, CA, USA) at 440 nm according to the manufacturer's instructions.

### 2.6. Evaluation of Apoptosis

The TUNEL staining was carried out according to the manufacturer's instructions (Roche, Basel, Switzerland). TUNEL-positive cells were analyzed using a flow cytometer (BD, C6, USA). Results were expressed as relative change vs control. Activities of caspase 3 and caspase 9 were determined using commercial kits (BioVision, USA).

### 2.7. Real-Time PCR

Total RNA was isolated from cells using Trizol reagent (Invitrogen, USA). NanoDrop ND-2000 was used to qualify the RNA. Revert Aid First Strand cDNA Synthesis Kit (Thermo Scientific, USA) was used to synthesize cDNA according to the manufacturer's protocols. RT-qPCR was performed by using the LightCycler 480 SYBR Green I Master kit (Roche, Basel, Switzerland) on a Bio-rad CFX96 Detection System (Bio-rad, USA). *β*-Actin was used as an internal control. 2^−ΔΔCt^ method was used to evaluate the level of mRNA.

### 2.8. Western Blotting

Cells were collected and lysed on ice. After centrifugation, protein concentration was measured using a BCA protein assay kit (ThermoScientific, USA). Equivalent amounts of protein (2–25 *μ*g) onto 10–15% SDS-polyacrylamide gels. Then, protein was transferred onto a PVDF membrane and blocked with 5% bovine serum albumin solution for 1 h at room temperature. The membranes were then probed with primary antibodies (1 : 1000 dilution in 5% BSA in TBS) overnight at 4°C. Following the incubation, the membranes were washed using TBST and incubated with HRP (Horseradish peroxidase)-conjugated secondary antibodies (Santa Cruz Biotechnology, USA) for 1 h at room temperature. After washing, protein bands were detected with an enhanced chemiluminescence detection kit (Pierce, Rockford, lL, USA). Images were analyzed using Image J software (National Institutes of Health, Bethesda, MD, USA).

### 2.9. Evaluation of Oxidative Stress

The level of intracellular reactive oxygen species by the use of a fluorescent probe DCFH-DA. This molecule could cross cell membranes and can subsequently be hydrolyzed by intracellular esterase to nonfluorescent DCFH. After the experiment, cells were harvested and resuspended in serum-free medium containing 10 *μ*M DCFH-DA and incubated at 37°C in the dark for 30 min. After three-time washing, cells were analyzed using a flow cytometer. Malondialdehyde (MDA) content, superoxidase dismutase (SOD) activity, glutathione (GSH) level, and glutathione disulfide (GSSG) level were determined using commercial assay kits (Biovision, USA) according to the manufacturer's protocols as previously reported [16–18].

### 2.10. DNA Copy Number

Relative mtDNA copy number was evaluated using real-time PCR to assess the ratio of COX I to GAPDH. In brief, genomic DNA was isolated using the E.Z.N.A.™ DNA Isolation Kit (Omega Biotek Inc, USA) following the manufacturer's protocols. The primer sequences for PCR were as follows: COX I: forward: 5′-TCGCCATCATATTCGTAGGAG-3′; reverse: 5′-GTAGCGTCG TGGTATTCCTGA- 3′; GAPDH: forward: 5′-GAGGGGCCATCCACAGTCTTC-3′; reverse:5′- CATCACCATCTTCCAGGAGCG- 3′. RT-qPCR was performed by using the LightCycler 480 SYBR Green I Master kit (Roche, Basel, Switzerland) on a Bio-rad CFX96 Detection System (Bio-rad, USA).

### 2.11. Immunoprecipitation

Cells were lysed using a commercial lysis buffer (Beyotime, Shanghai, China). After centrifugation, the supernatant was obtained. 2 *μ*g P66shc antibody was incubated with a precleared supernatant for 12 h at 4°C. This was followed by additional incubation of protein A/G beads (Beyotime, Shanghai, China) for 12 h at 4°C. After washing, protein A/G beads were solubilized in 3X SDS sample buffer. The immunoprecipitated protein was collected for further western blot detection.

### 2.12. Statistical Analysis

Data were expressed as mean ± S.D. from at least three independent experiments. Statistical analysis was performed using Graph Pad Prism 6.0 software. Analysis of data was performed using a one-way analysis-of-variance (ANOVA) test, followed by Tukey's post hoc test. A difference was considered statistically significant at *p* < 0.05.

## 3. Results

### 3.1. KFL Ameliorated OGD-Induced Cytotoxicity in PC12 Cells

**Figure 1:**
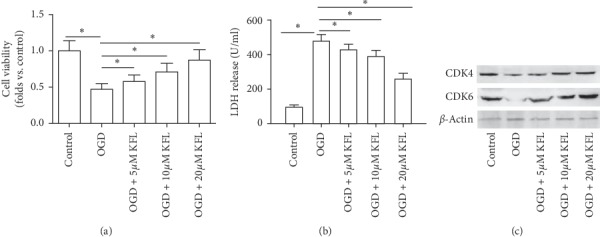
KFL ameliorated OGD-induced cytotoxicity in PC12 cells. (a) Cell viability was determined using the CCK8 assay kit. (b) LDH release in the medium was determined using a commercial kit. (c) Protein expression of CDK4 and CDK6 was determined using western blot. ^*∗*^*p* < 0.05.

In Figure 1(a), we found that OGD-induced decrease of cell viability in PC12 cells was significantly inhibited by KFL, which effect was in a concentration-dependent manner. KFL also inhibited OGD-induced increase of LDH release in cell culture medium (Figure 1(b)). In Figure 1(c), we showed that OGD resulted in a substantial decrease of CDK4 and CDK6 expression in PC12 cells, which was significantly inhibited by KFL. The results demonstrated that KFL attenuated OGD-induced cytotoxicity in PC12 cells, indicating a potential role of KFL in protecting against ischemic stroke.

### 3.2. KFL Ameliorated OGD-Induced Mitochondrial Apoptosis in PC12 Cells

**Figure 2:**
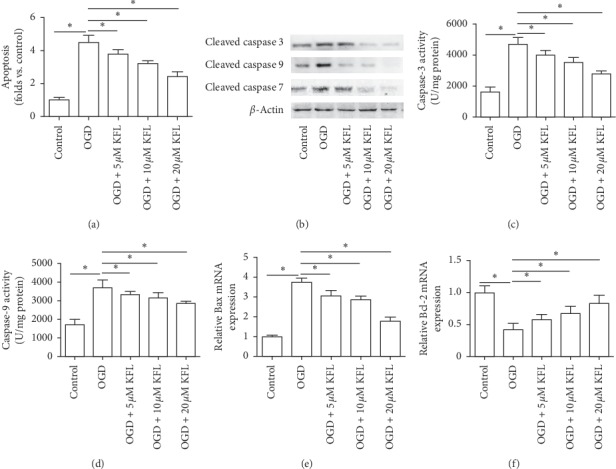
KFL ameliorated OGD-induced mitochondrial apoptosis in PC12 cells. (a) Apoptosis was measured using a TUNEL assay kit. (b) Protein expression of cleaved caspase 3, caspase 9, and caspase 7 was determined using western blot. (c and d) Caspase 3 and caspase 9 activities were measured using commercial kits. (e and f) mRNA expression of Bax and Bcl-2 was measured using real-time PCR. ^*∗*^*p* < 0.05.

Apoptosis has been believed to be critical for neuronal death in ischemic stroke and has been extensively studied [19]. OGD-induced increase of TUNEL-positive cells was notably inhibited by KFL treatment, which effect was in a concentration-dependent manner (Figure 2(a)). Protein expression of cleaved caspase 3, caspase 9, and caspase 7 was substantially increased by OGD and this effect was remarkably prohibited by KFL (Figure 2(b)). Moreover, OGD resulted in an increase of activities of caspase 3 and caspase 9 was inhibited by KFL (Figures 2(c) and 2(d)). Furthermore, OGD induced a significant increase of Bax mRNA expression and an obvious decrease of Bcl-2 expression in PC 12 cells (Figures 2(e) and 2(f)). The changes of Bcl-2-associated X (Bax) and B-cell lymphoma 2 (Bcl-2) mRNA expression induced by OGD were notably inhibited by KFL (Figures 2(e) and 2(f)). The results indicated that KFL could attenuate OGD-resulted apoptosis in PC12 cells.

### 3.3. KFL Ameliorated OGD-Induced Mitochondrial Dysfunction in PC12 Cells

**Figure 3:**
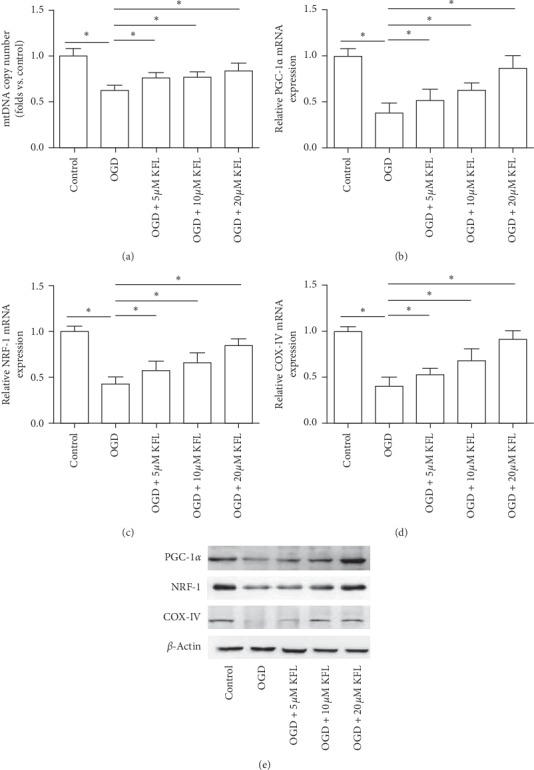
KFL ameliorated OGD-induced mitochondrial dysfunction in PC12 cells. (a) mtDNA copy number was measured using a commercial assay kit. (b–d) mRNA expression of PGC-1*α*, NRF-1, and COX-IV was measured using real-time PCR. (e) Protein expression of PGC-1*α*, NRF-1, and COX-IV was measured using western blot. ^*∗*^*p* < 0.05.

To further explore the effect of KFL on mitochondrial function in the context of OGD-induced injury in PC12 cells, we showed that KFL significantly increased the mtDNA copy number in the presence of OGD treatment (Figure 3(a)). Peroxisome proliferator-activated receptor gamma coactivator 1-alpha (PGC-1*α*) and nuclear respiratory factor-1 (NRF-1) are central controllers of mitochondrial biogenesis [20, 21]. Our results showed that OGD-resulted decrease of mRNA and protein expression of PGC-1*α* and NRF-1 were significantly inhibited by KFL (Figures 3(b)–3(e)). Moreover, the decrease of cytochrome c oxidase (COX)-IV mRNA and protein expression induced by OGD was inhibited concentration-dependently by KFL (Figures 3(d) and 3(e)). The data suggested that KFL ameliorated OGD-induced mitochondrial dysfunction in PC12 cells.

### 3.4. KFL Ameliorated OGD-Induced Oxidative Stress in PC12 Cells

**Figure 4:**
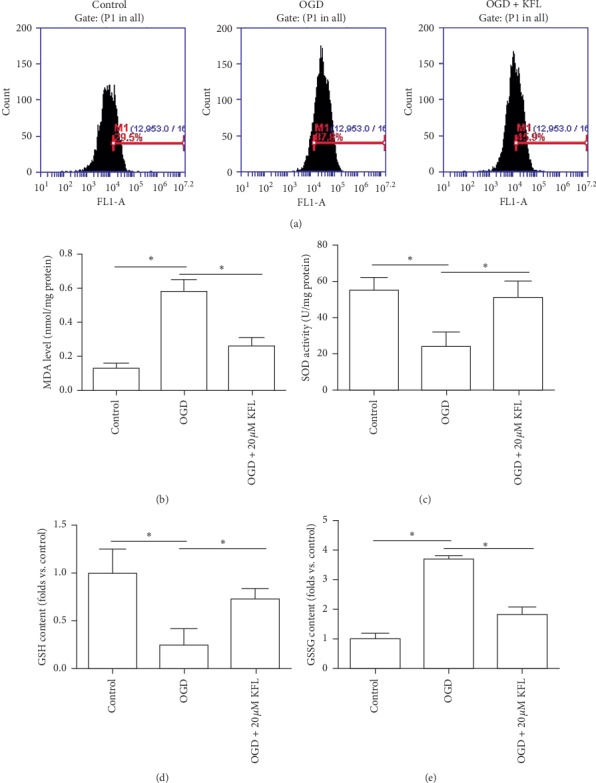
KFL ameliorated OGD-induced oxidative stress in PC12 cells. (a) ROS level was measured using an oxidant sensitive probe. (b–e) MDA content, SOD activity, GSH content, and GSSG content were determined using commercial kits. ^*∗*^*p* < 0.05.

Oxidative stress is an important pathophysiological condition that is associated with apoptosis, mitochondrial dysfunction, and ischemia and reperfusion injury [22]. In the study, we further studied whether KFL played a role in the regulation of reactive oxygen species (ROS) and antioxidant defense under OGD condition. As shown in Figure 4(a), we found that the number of cells with positive ROS-probe was significantly increased by OGD. KFL showed an obvious inhibition on OGD-induced increase of ROS level (Figure 4(a)). OGD-induced increase of MDA content in PC12 cells was significantly inhibited by KFL (Figure 4(b)). The decrease of SOD activity and GSH content induced by OGD was significantly inhibited by KFL (Figures 4(c) and 4(d)). GSSG content was increased by OGD and this increase was inhibited by KFL (Figure 4(e)). The results indicated that OGD induced significant oxidative stress and KFL inhibited this oxidative status in PC12 cells.

### 3.5. Downregulation of P66 Was Involved in the Neuroprotective Effects of KFL Against OGD in PC12 Cells

**Figure 5:**
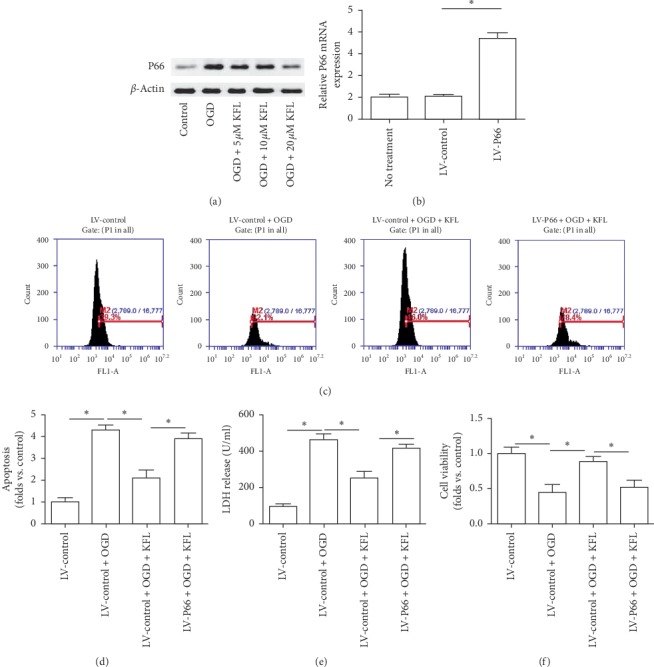
Downregulation of P66 was involved in the neuroprotective effects of KFL against OGD in PC12 cells. (a) Protein expression of P66shc was determined using western blot. (b) The efficiency of LV-P66 transfection was confirmed by real-time PCR. (c) ROS level was measured using an oxidant sensitive probe. (d) Apoptosis was measured using a TUNEL assay kit. (e) LDH release in medium was determined using a commercial kit. (f) Cell viability was determined using the CCK8 assay kit. ^*∗*^*p* < 0.05.

We further designed experiments to study the mechanism of KFL-induced inhibition of oxidative stress in OGD-treated PC12 cells. As shown in Figure 5(a), we found that OGD induced a significant increase of P66shc expression. The treatment of KFL significantly inhibited the protein expression of P66shc in OGD-treated PC12 cells. P66shc is considered to be a pivotal regulator of redox balance through promoting ROS generation [23]. To test whether the regulation of P66shc was involved in KFL-induced protective effects, we upregulated the mRNA expression of P66shc using lentivirus infection (Figure 5(b)). As shown in Figure 5(c), the upregulation of P66shc inhibited the suppressive effect of KFL on ROS generation in OGD-treated PC12 cells. Moreover, the effect of KFL on apoptosis, LDH release, and cell viability was inhibited by the upregulation of P66shc (Figures 5(d)–5(f)). The results suggested that the downregulation of P66shc was involved in the protective effect of KFL against OGD-induced injury in PC12 cells.

### 3.6. Upregulation of SIRT1 Was Involved in the Neuroprotective Effects of KFL Against OGD in PC12 Cells

**Figure 6:**
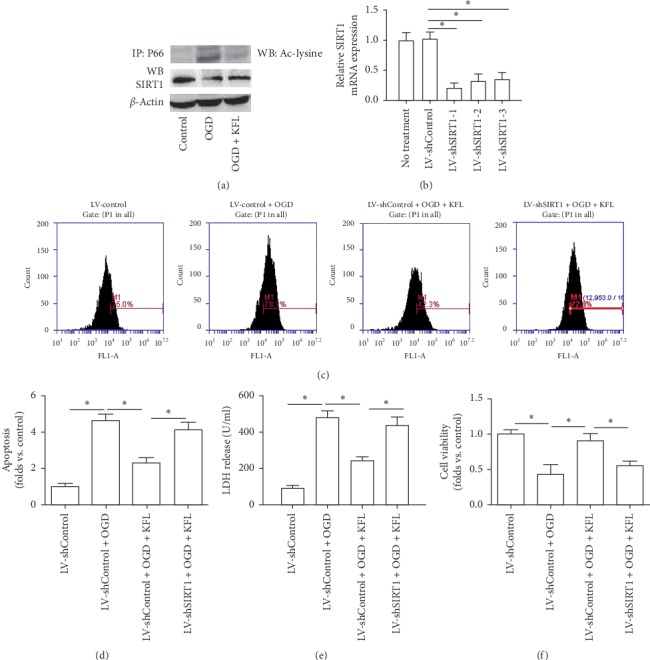
Upregulation of SIRT1 was involved in the neuroprotective effects of KFL against OGD in PC12 cells. (a) Cell lysates were extracted and subjected to immunoprecipitation with an anti-P66shc antibody. The precipitates were then evaluated with an antiacetylated-lysine (Ac-lysine) antibody. Protein expression of SIRT1 was determined using western blot. (b) The efficiency of LV-shSIRT1 transfection was confirmed by real-time PCR. (c) ROS level was measured using an oxidant sensitive probe. (d) Apoptosis was measured using a TUNEL assay kit. (e) LDH release in medium was determined using a commercial kit. (f) Cell viability was determined using the CCK8 assay kit. ^*∗*^*p* < 0.05.

Furthermore, we explored the possible mechanism of KFL-induced regulation of SIRT1. In Figure 6(a), we found that OGD resulted in a significant increase of acetylated form of P66shc, which effect was nearly abolished by KFL. In addition, OGD induced an obvious reduction of SIRT1 expression and this effect was inhibited by KFL. Previous studies have reported that deacetylation of P66shc by SIRT1 is an important physiological regulatory mechanism [24]. To further evaluate the involvement of SIRT1 in KFL-induced beneficial effects, we knocked down the expression of SIRT1 using lentivirus vectors (Figure 6(b)). Since LV-shSIRT1-1 showed the most significant efficiency of downregulation, we used LV-shSIRT1-1 in the following experiments. As shown in Figure 6(c), the downregulation of SIRT1 inhibited the suppressive effect of KFL on ROS generation in OGD-treated PC12 cells. Moreover, the effect of KFL on apoptosis, LDH release, and cell viability was inhibited by the downregulation of SIRT1 (Figures 6(d)–6(f)). The results suggested that the upregulation of SIRT1 was involved in the neuroprotective effects of KFL against OGD in PC12 cells.

## 4. Discussion

It has been believed that KFL, a phytoestrogen that belongs to the family of flavonoids, has various activities such as antioxidant, anti-inflammatory, antidiabetic, and anticancer activities [10, 11, 25]. More and more attention has been paid to the protective effects of KFL in the neural system. Chitturi et al. found that KFL exhibited beneficial effects after developmental traumatic brain injury and showed effects on the regulation of mitochondrial function, oxidative metabolism, and neural viability [26]. Kim et al. and colleagues have provided evidence of the protective effects of KFL and its derivatives on cognitive activities [12]. It was also shown that KFL glycosides attenuated brain injury and neuroinflammation in transient focal stroke [13]. KFL could inhibit extrasynaptic NMDAR-mediated downregulation of TRk*β* in rat hippocampus during hypoxia [27]. KFL was also shown to afford protection against ischemia/reperfusion-induced damage in rat brain [14]. However, the mechanism of KFL-induced neuronal protection is still largely not understood.

In the current study, we aimed to investigate the probable mechanism of KFL-induced effect on ischemia/reperfusion injury in PC12 cell model in vitro. We used OGD- reoxygenation to mimic ischemia/reperfusion administration in vitro. We showed that KFL could significantly inhibit OGD-induced decrease of cell viability, mitochondrial dysfunction, mitochondrial apoptotic pathway, and apoptosis. All these results suggested that KFL exhibited protective effects against OGD-induced cytotoxicity in PC12 cells.

The effect of KFL on the process of apoptosis has been demonstrated to be paradoxical [28–30]. Guo et al. showed that KFL induced apoptosis in HepG2 cells via activation of the endoplasmic reticulum stress pathway, exhibiting an antitumor effect [30]. Che et al. reported that KFL could alleviate ox-LDL-induced apoptosis by upregulation of autophagy via inhibiting PI3K/Akt/mTOR pathway in human endothelial cells [28]. We showed that KFL exhibited a significant inhibitory effect on OGD-induced mitochondrial apoptosis in PC12 cells. The differential effect of KFL on apoptosis may be dependent on different cell types and concentrations.

Redox signaling was closely associated with mitochondrial function and apoptotic pathway. In this study, we also found that OGD-induced oxidative stress was significantly inhibited by KFL, suggesting a substantial role of KFL in the redox regulation under OGD condition. P66 isoform of SHC1 (p66shc) proapoptotic ROS-elevating SHC family adaptor is an important regulator of redox homeostasis [31]. Genetic ablation of the p66Shc has been shown to reverse cognitive deficits and improve mitochondrial function in an APP transgenic mouse model of Alzheimer's disease [32]. In the present study, we found that OGD significantly increased total protein expression and acetylated form of P66shc, which was involved in the ROS generation and activation of mitochondrial apoptosis and cytotoxicity. KFL treatment ameliorated OGD-induced PC12 cell injury through inhibition of total protein expression and acetylated form of P66shc. Previous reports have shown that acetylation of P66shc is critical for its effect on ROS generation and SIRT1 is an important regulator of the acetylation of P66shc [24]. To further examine the possible mechanism of KFL-induced regulation of P66shc, we examined the role of SIRT1. We showed that KFL could inhibit OGD-induced decrease of SIRT1 expression and downregulation of SIRT1 suppressed KFL-induced protective effects. The data suggested that SIRT1-mediated regulation of P66shc may be involved in OGD-induced injury in PC12 cells and the SIRT1/P66shc pathway may be a major target of KFL.

**Figure 7:**
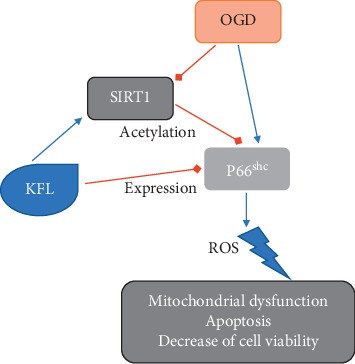
Schematic figure of KFL-induced protective effects against OGD-induced injury in PC12 cells.

In summary, we identified that KFL exhibited a beneficial effect against OGD-induced cytotoxicity in an ischemia/reperfusion injury cell model. KFL inhibited mitochondrial dysfunction, apoptosis, and oxidative stress in OGD-treated PC12 cells. KFL-induced protein expression of SIRT1 and inhibition of P66shc expression and acetylation is critical for its protective effects (Figure 7). The findings suggest that KFL may be a promising choice for the intervention of ischemic stroke and highlighted the SIRT1/P66shc signaling.

## Data Availability

The data used to support the findings of this study are available from the corresponding author upon request.
